# Interpretation of the hygiene and microflora hypothesis for allergic diseases through epigenetic epidemiology

**DOI:** 10.4178/epih.e2018006

**Published:** 2018-03-10

**Authors:** Jong-Myon Bae

**Affiliations:** Department of Preventive Medicine, Jeju National University School of Medicine, Jeju, Korea

**Keywords:** Gene-environment interaction, Epigenetics, Epidemiology, Allergy and immunology, Gastrointestinal microbiome

## Abstract

The hygiene hypothesis (HH) proposed by Strachan in 1989 was expanded to explain the inverse association between the occurrence of allergy disorders and the risk of infectious diseases and parasite infestation. The microflora hypothesis (MH) suggests that gut microbial dysbiosis in early life might trigger hypersensitivity disorders. The sharing concept of both HH and MH is gene-environment interaction, which is also a key concept in epigenetics. The amalgamation of epidemiology and epigenetics has created a scientific discipline termed epigenetic epidemiology. To accomplish an era of gene-environment-wide interaction studies, it is necessary to launch a national human epigenome project.

## CLASSIC EPIDEMIOLOGICAL INTERPRETATION OF THE HYGIENE HYPOTHESIS

Based on the results that repeated exposures to infection in early life after birth led to fewer incidences of hypersensitive immune diseases, Strachan [1] proposed the hygiene hypothesis (HH) in 1989. Epidemiological evidence that supports HH can be summarized into two categories [2]. First, there is a geographically inverse distribution between the levels of various infective diseases, including parasites and the prevalence of allergy/autoimmune diseases [3]. Second, an immigrant study showed that the prevalence of immune disease increased in the second generation after moving to a country with a high prevalence of diseases [4]. In addition, the experimental functions of HH were summarized to five and four mechanisms by Okada et al. [2] and Bach & Chatenoud [4], respectively (Table 1).

Thus, HH has been the major hypothesis to explain epidemiological phenomena, in which the prevalence of allergic diseases, including atopy, increases in addition to the improvement of hygiene after the Industrial Revolution and the environmental change brought about by vaccines that have reduced the risk of infection [2,5-9]. Furthermore, HH is applied to explain the trends in autoimmune diseases, such as type 1 diabetes mellitus, multiple sclerosis [3-5], and chronic inflammatory diseases, including inflammatory bowel disease and neuro-inflammatory disorder [7].

The epidemiology of HH shows that immune tolerance is induced by infection in early life, which leads to the reduction of immunity-related diseases [5]. Therefore, environmental changes, including lifestyle, which reduce the risk of infection during early life, affect the immune system [2,6,9-11]. In particular, the variation in the prevalence of immune disease based on rapid environmental changes is unable to be explained by genetic factors alone [6]; instead, the gene-environment interaction should be involved in the interpretation [9]. Hence, as it has been broadly interpreted that environmental changes in early life affect lifetime disease occurrence [12,13], the theory of ‘The Developmental Origins of Health and Disease’ (DoHaD) was proposed [12-14].

## EPIGENETIC INTERPRETATION OF THE HYGIENE HYPOTHESIS

Parasites, bacteria, and viruses were major targets of the studies of infectious agents that affect immunity in early life [5,7,10]. In addition, it has been known that microflora in the body, particularly, gut microbiota, as well as infectious agents, affect immunoregulation [5,8]; thus, the previous HH has been replaced with the microflora hypothesis (MH) [9,11,15]. To emphasize that MH is a new interpretation of HH, rather than a new hypothesis, HH was considered an alias as the ‘old friends hypothesis’, which focuses on parasitic infection in classic epidemiology [5].

According to MH, the immune regulatory function is affected by gut microbial dysbiosis that is caused by feeding (breast feeding vs. bottle feeding), childbirth type (vaginal delivery vs. cesarean section), and exposure to antibiotics [5,8,10,11].

In addition to MH, the previously mentioned DOHaD theory suggested that the genotype was predetermined by fertilization, but that the disease risk was determined by the exposure to external environments in early life; therefore, studies related to this theory correspond to epigenetic studies [16-18]. Epigenetics refers to ‘heritable changes in gene expression not caused by changes in the DNA sequence’, which was first used by Conrad Hal Waddington in approximately 1950 [19]. Although there is no change in the genetic information of DNA, the mechanisms to induce epigenetic changes include DNA methylation, histone modification, and microRNAs [20]. Thus, in 2007, Bird [21] proposed to redefine epigenetics as ‘the structural adaptation of chromosomal regions’.

Currently, the epigenetic interpretation of allergic diseases can be summarized as follows: the incidence of diseases results from (1) epigenetic alterations by exposure to various environmental factors; and (2) epigenetic modification of mediators that function for genetic sensitivity [17,18]. In such trends, epigenetic epidemiology study has been performed to understand the occurrence of common complex diseases with respect to gene-environment interactions, which takes advantages of epidemiology to identify influential environmental factors and epigenetics, which can reveal cellular and molecular mechanisms [22,23].

## SUGGESTION OF EPIGENETIC EPIDEMIOLOGY

Waterland & Michels [24] defined epigenetic epidemiology as ‘the study of the association between epigenetic variations and the risk of disease in humans’. Exposure factors that cause epigenetic variations include the lifestyle of parents, such as smoking, alcohol consumption, and diet [25], and the exposure to various physical, chemical, biological, and social environments during early life [14, 16,26,27]. The resulting diseases include allergies and autoimmune diseases, as well as various complex diseases such as cancer, diabetes mellutus, obesity, arteriosclerosis, autism, and mental diseases, et cetera [17,20,22]. Furthermore, the inter-generational transmission phenomenon that inherits diseases resulted from epigenetic alteration should become another subject of study [14].

As epigenetic alteration by environmental exposure differs depending on age, studies of epigenetic epidemiology need to secure age-matched controls [22]. In addition, a cohort study is required to repeatedly gather samples to examine variations and to assess them over the long term [23,27].

As such, epigenetic epidemiology can reveal whether an epigenetic alteration is a causal factor, biomarker, or modifier of a specific disease, which then can be utilized for preventive treatment, early diagnosis, and the intervention treatment of the disease [5, 17,23], respectively. As it evaluates the relationship between epigenetic alteration and the disease risk for each individual, epigenetic epidemiology is directly linked with personalized medicine [15].

Recently, An [28] emphasized the necessity of the national genome study. Given the burden of cancer and cardiovascular diseases, which represents the major causes of mortality in Korea, a nationwide epigenetic epidemiology study is of great importance. Moreover, the sample types, collection timing, and sample size for epigenetic epidemiology studies are significantly different from those of the genomic epidemiology studies [20,26], so that a foundation for the study of epigenetic epidemiology should be newly established in Korea, which would facilitate gene-environment-wide interaction studies [29,30], the ultimate goal of epidemiology.

## Figures and Tables

**Table 1.** Mechanisms of the hygiene hypothesis

Study	
Okayda et al. (2010) [2]	Bach et al. (2012) [4]
Th1-Th2 deviation	Identification of infectious agents and their protective constituents
Antigenic competition/ homeostasis	Role of anti-infectious immune responses on lymphocyte homeostasis and immunoregulation
Immuno-regulation	Stimulatory role of toll-like receptors
Non-antigenic ligands	Other mechanisms
Gene-environment interactions	

Th, T helper type.

## SUPPLEMENTARY MATERIALS

Supplementary Material: Korean version is available at http://www.e-epih.org/.
