# Supplementary material for: Interpretation of the hygiene and microflora hypothesis for allergic diseases through epigenetic epidemiology
Source: Epidemiol Health. 2018 Mar 10;40:e2018006. doi: 10.4178/epih.e2018006 (PMC5968202; doi:10.4178/epih.e2018006)
Supplement: Supplementary file 1 [file epih-40-e2018006-supplementary.pdf]

<Perspective>

알레르기 질환에 대한 위생 및 체내 미생물 가설의 후성유전역학적 해석

Interpretation the Hygiene and Microflora Hypothesis for Allergic Diseases through Epigenetic Epidemiology

배종면

국립제주대학교 의학전문대학원 예방의학교실

1. 위생가설의 고전역학적 해석

Strachan [1]은 출생 후 성장 초기에 감염기회가 높을수록 과민성 면역질환이 덜 생긴다는 결과를 근거로 1989년에 위생가설 (Hygiene hypothesis, 이하 HH)을 제기하였다. 이후 이를 지지하는 역학적 근거들을 다음 2가지로 요약할 수 있다 [2]. 첫째는 기생충을 포함한 여러 감염병 수준과 알레르기/자가면역질환의 유병수준 간에 지리학적으로 반비례 분포를 보인다는 점이며 [3], 둘째는 면역질환의 유병률이 높은 나라로 이주했을 때 이민 2세에서 유병률이 높아진다는 이민자 연구 결과이다 [4]. 그리고 HH의 실험실적 기전에 대하여 Okada et al. [2]는 5가지로, Bach & Chatenoud [4]는 4가지로 정리하여 제시하고 있다 (표 1)

이에 따라 HH는 산업혁명 이후 위생 개선과 감염 예방용 백신 접종으로 출생 후 성장과정에서 감염의 기회가 줄어드는 환경 변화에 따라 아토피를 포함한 알레르기성 질환의 유병률이 더 높아지는 역학적 현상을 설명하는 주된 가설이 되어 왔다 [2,5-9]. 나아가 제1형 당뇨 (Type 1 diabetes mellitus)나 다발성 경화증 (multiple sclerosis) 같은 자가면역질환 (autoimmune diseases) [3-5] 뿐만 아니라 염증성 장질환 (inflammatory bowel disease), 신경염증 질환 (neuro-inflammatory disorder) 등의 만성염증질환 [7]의 발생 추세에 대하여도 HH를 적용하고 있다.

이상의 HH 가설이 담고 있는 역학적 의미는 생애 초기의 감염으로 면역 내성 (immune tolerance)이 생기며, 이에 따라 면역관련 질환의 발생이 감소한다는 것이다 [5]. 다시 말해서 성장 초기에 감염의 위협을 낮추는 생활습관 (lifestyle)을 포함한 환경의 변화는 면역체계에 영향을 미친다는 의미이다 [2,6,9-11]. 특히 급격한 환경변화에 따른 면역질환의 유병률 변동 현상은 유전적 소인만으로는 설명할 수 없으며 [6], 유전-환경의 상호작용 (Gene-Environment interaction)이 관여한다는 것을 의미한다 [9]. 이에 따라 성장초기의 환경 변화가 생애 질병발생에 영향을 미친다고 확대 해석이 되면서 [12,13], 'The Developmental Origins of Health and Disease' (DoHaD) 이론이 제기되었다 [12-14].

2. 위생가설의 후성체학적 해석

생애 초기의 면역에 영향을 미치는 감염원으로 기생충, 세균, 바이러스 등이 주된 연구대상이 되었다 [5,7,10]. 이후 체외 감염원 뿐만 아니라 체내 미생물 – 특히 장내 미생물군 (gut microbiota) -이 면역조절 (immuno-regulation)에 영향을 미친다는 사실들이 알려지면서 [5,8], 기존의 HH는 '체내미생물 가설' (Microflora Hypothesis, 이하 MH) 로 대치되었다 [9,11,15]. MH가 HH와 전혀 다른 새로운 것이 아니라 기존의 HH에 대한 새로운 해석이라는 의미를 강조하기 위하여, 고전역학 (classic epidemiology)에서 기생충 감염에 초점을 둔 HH를 'old friends hypothesis'란 별칭이 제시되었다 [5].

MH을 요약하면, 생애 초기에 수유 (모유 대 분유), 출생양식 (질식분만 대 제왕절개 분만), 항생제 노출 여부 등으로 장내 미생물 균주가 비정상적으로 변화하면서 (gut microbial dysbiosis), 면역 조절 기능에 영향을 준다는 것이다 [5,8,10,11].

MH 뿐만 아니라 앞서 언급한 DOHaD 이론은 수정 (fertilization)으로 유전자형이 이미 결정된 이후 성장 초기에 노출되는 외부 환경에 따라 질병 발생 위험이 결정된다는 점에서, 해당 이론과 관련한 연구들은 후성유전학 (Epigenetics) 연구에 해당한다 [16-18]. 후성유전학이란 DNA 변화 없이 유전가능한 발현 변화가 생기는 것 (heritable changes in gene expression not caused by changes in the DNA sequence)이란 의미로 Conrad H Waddington이 1950년경에 처음 사용한 용어이다 [19]. 유전정보를 담고 있는 DNA는 변함이 없는 상황에서 후성유전학 현상이 생기는 기전으로는 DNA 메틸화 (DNA methylation), 히스톤 변형 (Histone modification), 소RNA (microRNA) 등이 알려져 있다 [20]. 이에 따라 2007년 Bird [21]는 후성유전학을 염색체 부위의 구조적 적응 (the structural adaptation of chromosomal regions)으로 재정의할 것을 제안하였다.

현재 시점에서 알레르기 질환에 대한 후성유전학 해석은 (1) 각종 환경요소의 폭로로 인한 후성유전적 변형과 (2) 유전자 감수성의 조정자 (mediator) 역할을 하는 후성유전

적 변형에 의해 질병발현 여부가 결정된다고 요약할 수 있다 [17,18]. 이런 흐름 속에서, 주요복합질병 (common complex diseases)의 발생으로 이해하는 유전-환경의 상호작용에 있어, 어떤 환경요소가 영향을 미치는가를 알아내는 역학 (epidemiology)과 세포 분자 수준에서 그 기전을 밝히는 후성유전학 (epigenetics)의 장점을 모두 살린 후성유전역학 (Epigenetic Epidemiology) 연구가 진행되어 오고 있다 [22,23].

3. 후성유전역학의 제언

Waterland & Michels [24]은 후성유전역학을 '후성유전적 변이에 따른 질병발생위험 여부를 연구하는 학문' (the study of the association between epigenetic variation and the risk of disease in human)이라고 정의하였다. 후성유전적 변이를 야기하는 폭로요인으로는 흡연, 음주, 식이 등의 부모의 생활습관 [25] 뿐만 아니라, 생애초기에 노출되는 각종 물리-화학-생물-사회 환경에 관한 것들이다 [14,16,26,27]. 그리고 결과에 해당하는 질병으로는 알레르기 및 자가면역질환 뿐만 아니라 암, 당뇨, 비만, 동맥경화증, 자폐증, 정신질환 등의 각종 복합질환들이 망라되고 있다 [17,20,22]. 더 나아가 후성적 변이로 발생한 질병이 다음 세대로 유전되는 inter-generational transmission 현상도 연구의 대상이다 [14].

환경 폭로로 인한 후성유전적 변이는 연령에 따라 차이가 난다는 점에서 후성유전역학연구 수행에 있어 환자와 연령을 짝지은 대조군 (age-matched controls)을 확보할 필요

성이 있다 [22]. 또한 변이 여부를 확인하기 위한 검체를 반복적으로 확보하고 장기간 측정할 필요성 때문에 코호트 연구가 필요하다 [23,27].

이렇게 후성유전역학으로 후성유전적 변이가 특정 질병의 원인적 요소 (causal factor), 생물지표 (biomarker), 조정 변수 (modifier) 인가에 따라 해당 질병의 예방조치, 조기 진단, 개입 치료에 각각 활용할 수 있다 [5,17,23]. 이런 활용을 위하여 개인별로 후성유전적 변이 여부와 질병 발생 위험간의 관련성을 평가한다는 점에서, 후성유전역학은 맞춤의학 (personalized medicine)과 직결된다 [15].

최근 Ah JY [28]는 국가 유전체 연구의 당위성을 역설하였는데, 한국인의 주요사망원인인 암과 심혈관계질환의 부담을 고려할 때 국가 단위의 후성유전역학연구의 당위성이 더욱 강조된다. 더군다나 후성유전역학 연구를 위한 검체 종류와 채취 시점, 규모 등에서 유전체역학연구와 큰 차이가 있다는 점에서 [20,26], 국내의 후성유전역학 연구를 위한 기반을 새로이 구축해야 한다. 그래야만 역학이 궁극적으로 추구하는 유전-환경 상호작용의 연구 (gene-environment-wide interaction studies, GEWIS) [29,30]를 해낼 수 있기 때문이다.

국문요약

1989년 제시된 위생가설 (Hygiene hypothesis)은 최근 체내미생물가설 (Microflora hypothesis) 대치되어 알레르기 질환의 발생기전을 해석하고 있다. 두 가설 모두 유전-환경 상호작용 gene-environment interaction) 을 강조하고 있다는 점에서 향후 후성유전역학 연구에 대한 활성화가 필요하다.

중심어: 유전-환경 상호작용, 후성유전학, 역학, 알레르기와 면역

References

1. Strachan DP. Hay fever, hygiene, and household size. BMJ. 1989;299(6710):1259-60.
2. Okada H, Kuhn C, Feillet H, Bach JF. The 'hygiene hypothesis' for autoimmune and allergic diseases: an update. Clin Exp Immunol. 2010;160(1):1-9.
3. Bach JF. The effect of infections on susceptibility to autoimmune and allergic diseases. N Engl J Med. 2002;347(12):911-20.
4. Bach JF, Chatenoud L. The hygiene hypothesis: an explanation for the increased frequency of insulin-dependent diabetes. Cold Spring Harb Perspect Med. 2012;2(2):a007799.
5. Stiemsma LT, Reynolds LA, Turvey SE, Finlay BB. The hygiene hypothesis: current perspectives and future therapies. Immunotargets Ther. 2015;4:143-57.
6. Bloomfield SF, Stanwell-Smith R, Crevel RW, Pickup J. Too clean, or not too clean: the hygiene hypothesis and home hygiene. Clin Exp Allergy. 2006;36(4):402-25.
7. Rook GA. Review series on helminths, immune modulation and the hygiene hypothesis: the broader implications of the hygiene hypothesis. Immunology. 2009;126(1):3-11.
8. Versini M, Jeandel PY, Bashi T, Bizzaro G, Blank M, Shoenfeld Y. Unraveling the Hygiene Hypothesis of helminthes and autoimmunity: origins, pathophysiology, and clinical applications. BMC Med. 2015;13:81.

9. Martinez FD. The coming-of-age of the hygiene hypothesis. Respir Res. 2001;2(3):129-32.

10. MacGillivray DM, Kollmann TR. The role of environmental factors in modulating immune responses in early life. Front Immunol. 2014;5:434.

11. Stiemsma LT, Turvey SE. Asthma and the microbiome: defining the critical window in early life. Allergy Asthma Clin Immunol. 2017;13:3.

12. Fleming TP, Velazquez MA, Eckert JJ. Embryos, DOHaD and David Barker. J Dev Orig Health Dis. 2015;6(5):377-83.

13. Braun MM, Ahlbom A, Floderus B, Brinton LA, Hoover RN. Effect of twinship on incidence of cancer of the testis, breast, and other sites (Sweden). Cancer Causes Control. 1995;6(6):519-24.

14. Miller M, Bailey B, Govindarajah V, Levin L, Metzger T, Pinney SM, et al. A community survey on knowledge of the impact of environmental and epigenetic factors on health and disease. Perspect Public Health. 2016;136(6):345-352.

15. Nicholson JK, Holmes E, Wilson ID. Gut microorganisms, mammalian metabolism and personalized health care. Nat Rev Microbiol. 2005;3(5):431-8.

16. Winett L, Wallack L, Richardson D, Boone-Heinonen J, Messer L. A Framework to Address Challenges in Communicating the Developmental Origins of Health and Disease. Curr Environ Health Rep. 2016;3(3):169-77.

17. Potaczek DP, Harb H, Michel S, Alhamwe BA, Renz H, Tost J. Epigenetics and allergy: from basic mechanisms to clinical applications. Epigenomics. 2017;9(4):539-571.

18. Kuriakose JS, Miller RL. Environmental epigenetics and allergic diseases: recent advances. Clin Exp Allergy. 2010;40(11):1602-10.

19. Slack JM. Conrad Hal Waddington: the last Renaissance biologist? Nat Rev Genet. 2002;3(11):889-95.

20. Riancho J, Del Real A, Riancho JA. How to interpret epigenetic association studies: a guide for clinicians. Bonekey Rep. 2016;5:797.

21. Bird A. Perceptions of epigenetics. Nature. 2007;447(7143):396-8.

22. Michels KB. Epigenetic epidemiology.New York:Springer; 2012.p.1-20.

23. Bakulski KM, Fallin MD. Epigenetic epidemiology: promises for public health research. Environ Mol Mutagen. 2014;55(3):171-83.

24. Waterland RA, Michels KB. Epigenetic epidemiology of the developmental origins hypothesis. Annu Rev Nutr. 2007;27:363-88.

25. Giordano C, Benyshek DC. DOHaD research with populations in transition: a case study of prenatal diet remote recall with Yup'ik Alaskan women. J Dev Orig Health Dis. 2015;6(2):79-87.

26. Foley DL, Craig JM, Morley R, Olsson CA, Dwyer T, Smith K, et al. Prospects for epigenetic epidemiology. Am J Epidemiol. 2009;169(4):389-400.

27. Ng JW, Barrett LM, Wong A, Kuh D, Smith GD, Relton CL. The role of longitudinal cohort studies in epigenetic epidemiology: challenges and opportunities. Genome Biol. 2012;13(6):246.

28. An JY. National human genome projects: an update and an agenda. Epidemiol Health 2017;39:e2017045.

29. Khoury MJ, Wacholder S. Invited commentary: from genome-wide association studies to gene-environment-wide interaction studies—challenges and opportunities. Am J Epidemiol. 2009;169(2):227-30; discussion 234-5.

30. Vercelli D. Gene-environment interactions in asthma and allergy: the end of the beginning? Curr Opin Allergy Clin Immunol. 2010;10(2):145-8.

Table 1. Mechanisms of the hygiene hypothesis

Okada et al. (2010) [2]	Bach & Chatenoud (2012) [4]
T helper type 1 (Th1)–Th2 deviation	Identification of Infectious Agents and Their Protective Constituents
Antigenic competition/homeostasis	Role of Anti-Infectious Immune Responses on Lymphocyte Homeostasis and Immunoregulation
Immuno-regulation	Stimulatory Role of Toll-Like Receptors
Non-antigenic ligands	Other Mechanisms
Gene-environment interactions	
